# Analysis on Differential Gene Expression Data for Prediction of New Biological Features in Permanent Atrial Fibrillation

**DOI:** 10.1371/journal.pone.0076166

**Published:** 2013-10-18

**Authors:** Feng Ou, Nini Rao, Xudong Jiang, Mengyao Qian, Wei Feng, Lixue Yin, Xu Chen

**Affiliations:** 1 School of Life Science and Technology, University of Electronic Science and Technology of China, Chengdu, China; 2 School of Electrical and Electronic Engineering, Nanyang Technological University, Nanyang Link, Singapore, Singapore; 3 Cardiovascular department, Sichuan Academy of Medical Sciences & Sichuan Provincial People's Hospital, Chengdu, China; University of Georgia, United States of America

## Abstract

Permanent Atrial fibrillation (pmAF) has largely remained incurable since the existing information for explaining precise mechanisms underlying pmAF is not sufficient. Microarray analysis offers a broader and unbiased approach to identify and predict new biological features of pmAF. By considering the unbalanced sample numbers in most microarray data of case - control, we designed an asymmetric principal component analysis algorithm and applied it to re - analyze differential gene expression data of pmAF patients and control samples for predicting new biological features. Finally, we identified 51 differentially expressed genes using the proposed method, in which 42 differentially expressed genes are new findings compared with two related works on the same data and the existing studies. The enrichment analysis illustrated the reliability of identified differentially expressed genes. Moreover, we predicted three new pmAF – related signaling pathways using the identified differentially expressed genes via the KO-Based Annotation System. Our analysis and the existing studies supported that the predicted signaling pathways may promote the pmAF progression. The results above are worthy to do further experimental studies. This work provides some new insights into molecular features of pmAF. It has also the potentially important implications for improved understanding of the molecular mechanisms of pmAF.

## Introduction

Atrial fibrillation (AF) is an extremely common cardiac rhythm disorder in population [1], [2]. The outstanding progresses have shown that AF pathophysiology involves electrical, structural and contractile remodeling, which is related to changes in cardiac gene expression.

Over the last decade, several studies have characterized the molecular basis of remodeling on a more global scale [3]–[7], in which Barth et al. [5] and Censi, et al. [6] respectively analyzed the same differential gene expression data with 10 permanent AF (pmAF) patients and 20 controls and found different biological features. In the atrial myocardium, Barth et al. [5] identified 1434 genes deregulated in pmAF. Besides, they found that most ventricular – predominant genes were upregulated in pmAF. They thought that dedifferentiation with adoption of a ventricular – like signature is a general feature of the fibrillating atrium. In the work of Censi, et al. [6], they applied the oblique principal component analysis (OPCA, a PCA – based method) to mine some new pmAF – related differentially expressed genes (DEGs) that were different from those in the work of Barth et al. [5] and found an attractor-like property of gene expression. Furthermore, Censi, et al. [7] analyzed the connection relationships among the identified DEGs. Their work showed that the connection relationships between AF and normal patient populations have great difference. However, any a quantitative analysis method for gene expression data has its limitations. For example, the PCA-based method might ignore some components that are, though statistically unimportant, biologically meaningful. The classical supervised approaches (such as SAM) were not able to well characterize different pathophysiological features of AF. Therefore, constant refinement is needed to evolve better methodologies to find more new biological features associated with AF for improved understanding of pathophysiological mechanisms of AF.

In this work, we designed an asymmetric principal component analysis (APCA) algorithm by considering the unbalanced sample numbers in most microarray data of case – control. From the data used by Barth et al. [5] and Censi, et al. [6], we identified 51 differentially expressed genes (DEGs) using the APCA algorithm, in which 42 DEGs are new findings compared with two related works and the existing studies. The enrichment analysis on GO and GAD showed that most identified DEGs are associated with the etiological factors inducing atrial fibrillation. Moreover, we predicted three new signaling pathways. Our analysis and the existing studies supported that the predicted signaling pathways may promote the pmAF progression. The obtained results in this work are worthy to do further experimental studies.

## Materials and Methods

### Data

The gene expression data used in this work were obtained from the public functional genomics data repository of the National Institute of Health (called Gene Expression Omnibus, GEO). The data with record #GSE2240 consists of samples from 30 patients, in which 10 patients had pmAF, whereas 20 patients had no history of AF and were in SR. The data are related to two Affymetrix platforms U133A and U133B. According to our statistics, 20995 of the 22283 genes in U133A have been annotated and the percentage of annotated genes is 94.2%. Only 14586 of the 22283 genes in U133B have been annotated and the annotated percentage is less than 65%. This may result in wrong interpretation to the final results. Therefore, our study focus on the data in U133A, which is a gene expression data set with 22283 rows (probes) and 30 columns (samples).

### APCA algorithm for identifying differentially expressed genes

The existing researches have shown that the PCA-based method on this data set succeed in discriminating patients from controls and in offering a mechanistic view relating AF condition to both cardiac muscle organization and inflammatory processes [6]. However, applying PCA-based method, the total covariance matrixes does not effectively remove the unreliable dimensions if one class is represented by its training data much better or much worse than the other class. The asymmetric principal component analysis (APCA) [8] and linear subspace learning-based dimensionality reduction [9] alleviate this problem by asymmetrically weighting the class conditional covariance matrices and by considering the important weak composition. Thus, we design an novel APCA-based method to identify the differentially expressed genes between patients with pmAF and the control group by combining the ideas in two works above [8], [9].

Assume that a gene expression dataset is represented by **X** with *m* rows (probes) and *l* columns (samples) and there is *m*>>*l*. The novel APCA – based method consists of four phases. In the phase one, we project **X** into a low dimension matrix **Xs**. The steps are as follows.

1. Construct **S** = **X** ^T^ **X**, where T denotes the transpose. Obviously, the size of **S** is *l*×*l*.
2. Apply PCA to **S** for obtaining the eigenvector matrix **EIV_S_** with size *l*×*l*, where one column is one eigenvector.
3. Compute **EIV_L_ = XEIV_S_**. The **EIV_L_** is the eigenvector matrix with size *m*×*l* corresponding to the nonzero eigenvalues of **XX** ^T^.
4. Project the **X** into **X_S_** through computing **Xs = EIV_L_** ^T^ **X**, which is of size *l*×*l*.

All principal components (PCs) and classification on Xs will be exactly the same with that on **X**. In phase two, we construct a new data matrix, shown as below.

1. Suppose that there are *n* control samples and *d* disease samples in the original data matrix **X**, and *n* + *d* = *l*. **Xs** is divided into two block matrices, that is, **X_S_** = [**X_Sn,_ X_Sd_**]. Thus, **X_Sn_** is of size *l*×*n* and **X_Sd_** *l*×*d*.
2. Calculate the mean vectors of **X_Sn_**, **X_Sd_** and **X_S_**, **M_Sn_**, **M_Sd_** and **M_S_** whose elements are the mean values over rows and whose sizes are all *l*×1.
3. Centralize the data matrices above by subtracting the means from **X_Sn_**, **X_Sd_** and **X_S_** to yield **Y_Sn_**, **Y_Sd_** and **Y_S_**.
4. Calculate the covariance matrices by **C_Sn_** = (1/*n*)**Y_Sn_Y_Sn_^T^** , **C_Sd_** = (1/*d*)**Y_Sd_Y_Sd_^T^** and **C_S_** = (1/*l*)[*n*(**M_Sn_**-**M_S_**)(**M_Sn_**-**M_S_**)^T^+*d*(**M_Sd_**-**M_S_**)(**M_Sd_**-**M_S_**)^T^] whose size are all *l*×*l*.
5. Construct a new matrix **C** = *a* **C_Sn_** + (1-*a*) **C_Sd_** +*b* **C_S_**, where the parameter *b* is a large value such as 20 and the parameter *a* is in the range of 0<*a*<1. They are determined according to the optimal classification result of patients/control samples based on the first two PCs.

In the third phase, we extract the principal components from **C** using the PCA algorithm [9]. The ‘shared variance’ is accounted for by the first PC and the minor PCs (from the second PC onward) keep trace of the relevant among sample difference [6]. In APCA, since the first two PCs could optimally classify the patients/control samples under the selected *a* and *b*, the second PC is used as the discriminant PC, which will be used to identify differentially expressed genes.

The discriminant PC is a vector with dimension of 22283×1, in which the value in each row represents the score of corresponding gene. In final phase, we identify the DEGs according to the scores of the genes. Those genes, whose scores (in absolute value) are higher than a given threshold (denoted by *θ*), are identified as the DEGs. As we all know, the selection of the threshold is very important. Many biologically relevant DEGs could not be identified if the threshold is too high, while lowering the identification threshold will increase the number of potential DEGs, including many false ones. Based on the reasons above, we select about half of the numerical range of the score *S* as the threshold.

Assuming that the score and its maximum value (in absolute value) are respectively indicated by *S* and |*S*_max_|, the numerical range of the score *S* is 0≤*S*≤|*S*_max_|, Thus, the threshold is set as *θ* = ⌊|*S*_max_|/2⌋.

### Prediction of pmAF – related pathways

The Affymetrix IDs of all the identified DEGs are entered into the KO-Based Annotation System (KOBAS) [10], which identifies the statistically enriched pathways. An identified pathway is predicted to be associated with pmAF when it satisfies the following criterion: the value of EASE (corrected P-value) for the pathway is less than or equal to 0.05 and meanwhile the number of DEGs involved in the pathway is larger than or equal to 2. This criterion ensures statistical credibility to the predicted results but not a consequence of chance.

### Calculation of ROC for the identified DEGs

In order to illustrate the reasonableness of threshold in our method as well as the reliability of the results, we assess the discrimination powers of identified DEG expression levels to classify normal and pmAF samples by calculating their receiver operating characteristic (ROC) curves and the areas under the curve (AUC). In ROC calculation, we respectively define pmAF and normal patients as positive and negative samples. Therefore, the true positive rate indicates how many correct positive results occur among all pmAF samples available during the test. False positive rate, on the other hand, represents how many incorrect positive results occur among all normal samples available during the test.

It is well known that pmAF is a kind of polygenic and multifactorial disease and so its occurrence and progress are associated with the combinational work of multiple genes. Thus, we calculate the ROCs and the AUCs of combinations among 51 identified DEGs. In order to find the combination rules of these DEGs, we analyze the connection relationships among them using correlation technique. Using the gene expression values of 51 DEGs across 10 samples in pmAF group of U133A, we calculate the correlation coefficient (CC) between each DEG pair. Under CC≥0.9 [7], we construct the connection relationship among 51 identified DEGs correspondent to pmAF patients and then combine these DEGs according to the connection relationship for conducting the calculation of ROCs and AUCs. For comparison, we also calculate the ROC and the AUC of each DEG for finding the discrimination powers of these DEGs individually.

## Results

### The discriminating PC

In U133A data set, there are respectively 22283 probes and 30 samples, which include 20 control samples and 10 disease samples. The independent number of control samples is 19 since the gene expression values of two samples in the control group are completely same. Thus, the total independent sample number is 29. The dimension of data matrix in our experiment are *m* = 22283, *l* = 29, *n* = 19 and *d* = 10.

**Figure 1:**
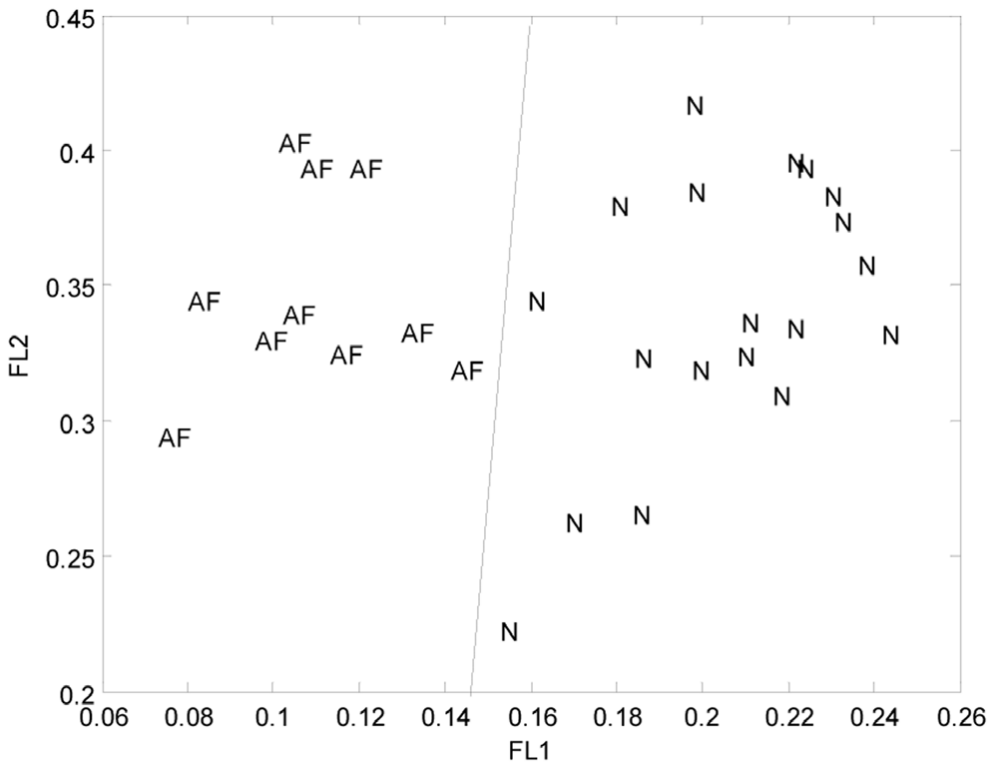
The classification results for the 29 samples by the first two PCs, where AF and N respectively indicate the pmAF and normal patients; The factor loading (FL) of a PC is defined as the correlation coefficients between original sample variables and this PC. FL1 and FL2 respectively denote the factor loadings of the first PC and the second PC on the 29 samples.

We let *b* be an integer of 20 and then uniformly sampled *a* over the interval (0, 1). Our simulation showed that two kinds of samples can be correctly classified by the first two PCs when taking *a* = 0.3 and *b* = 20 in the APCA algorithm, as shown in Figure 1. Table 1 gave the proportional and cumulative variances represented by the first 10 PCs under *a* = 0.3 and *b* = 20 when our method was applied to the U133A data set.

**Table 1:** Proportional and cumulative variances for the first 10 PCs.

Eigenvalue	Proportion	Cumulative
1	0.9961	0.9961
2	0.0020	0.9981
3	0.0006	0.9986
4	0.0003	0.9990
5	0.0002	0.9992
6	0.0001	0.9994
7	0.0001	0.9995
8	0.0001	0.9996
9	0.0001	0.9996
10	0.0001	0.9997

The results in the Table 1 show that the first PC accounts for 99.61% of total variability in both groups. With the large value of *b* (here *b* = 20), our algorithm ensures that the first PC is caused by the two class means and it represents the commonality between gene expression profiles on the genome-wide scale. Hence, all minor PCs are caused by the within-class variations of the both classes, which mainly concentrate on the second PC as shown in Table 1. The second PC captures the major difference between the two within-class variations of the two classes. Further, the pmAF patient and control samples are able to be correctly classified using the first two PC and so we select the second PC as the discriminating PC.

### The identified DEGs

We obtained each gene's score using the second PC in Table 1. The maximum value of the score is 11.02. Thus, the threshold of the score *θ* is calculated as 5. We identified 51 DEGs (corresponding to the 63 Affymetrix IDs) that have the scores (in absolute value) higher than the threshold. Their symbols, Affymetrix IDs (ID_REF), titles and scores are shown in Table 2, in which the genes DICER1, IGFBP2, LBH and NPR3 marked with italic are the differential expression genes previously reported in the work of [5] on the same data. Also, the genes IGFBP2, IGH@/IGHG1/IGHG2/IGHM/IGHV4-31, LOC100133662/RPS4Y1, RBP4, SFRP1 and XIST marked with bold are the DEGs previously found on the same data in the work of [6]. Compared with the results of two previous works, 42 new DEGs are identified by our novel method, which fully considers the important weak composition and so can identify some new genes that cannot be found by PCA algorithm and classical supervised approaches.

**Table 2:** Identified differential expression genes using the APCA algorithm.

No.	Gene symbol	ID_REF	Gene Title	Score
1	ADIPOQ	207175_at	adiponectin, C1Q and collagen domain	10.7946
			containing	
2	AMY1A /// AMY1B	208498_s_at	amylase, alpha 1A (salivary) /// amylase, alpha	5.5792
	/// AMY1C ///		1B (salivary) /// amylase, alpha 1C (salivary) ///	
	AMY2A /// AMY2B		amylase, alpha 2A (pancreatic) /// amylase, alpha	
			2B (pancreatic)	
3	BMP10	208292_at	bone morphogenetic protein 10	−9.7136
4	C2 /// CFB	202357_s_at	complement component 2 /// complement factor	7.3188
			B	
5	C3	217767_at	complement component 3	7.5830
6	CEBPA	204039_at	CCAAT/enhancer binding protein (C/EBP),	5.6280
			alpha	
7	COL21A1	208096_s_at	collagen, type XXI, alpha 1	7.4379
*8*	*DICER1*	*213229_at*	*dicer 1, ribonuclease type III*	*−5.0451*
9	DIRAS3	215506_s_at	DIRAS family, GTP-binding RAS-like 3	7.1738
10	EFEMP1	201843_s_at	EGF-containing fibulin-like extracellular matrix	5.7128
			protein 1	
11	FABP4	203980_at	fatty acid binding protein 4, adipocyte	11.0171
12	FHL2	202949_s_at	four and a half LIM domains 2	5.2915
13	GOLGA8A	208798_x_at	golgi autoantigen, golgin subfamily a, 8A	6.2200
14	HBA1 /// HBA2	204018_x_at	hemoglobin, alpha 1 /// hemoglobin, alpha 2	5.1573
		217414_x_at		5.2591
		211745_x_at		5.7071
		214414_x_at		5.7851
		209458_x_at		5.2950
		211699_x_at		5.3233
15	HBB	209116_x_at	hemoglobin, beta	5.4556
16	HP /// HPR	208470_s_at	haptoglobin /// haptoglobin-related protein	8.6784
		206697_s_at		7.9905
17	IGF1	209541_at	insulin-like growth factor 1 (somatomedin C)	6.1854
***18***	***IGFBP2***	***202718_at***	***insulin-like growth factor binding***	***5.4903***
			***protein 2*** **, 36 kDa**	
19	IGH@ /// IGHA1 ///	217022_s_at	immunoglobulin heavy locus /// immunoglobulin	6.4910
	IGHA2 ///		heavy constant alpha 1 /// immunoglobulin	
	IGHV3OR16-13 ///		heavy constant alpha 2 (A2m marker) ///	
	LOC100126583		immunoglobulin heavy variable 3/OR16-13	
			(non-functional) /// hypothetical LOC100126583	
**20**	**IGH@ /// IGHG1 ///**	**211430_s_at**	**immunoglobulin heavy locus ///**	**6.2667**
	**IGHG2 /// IGHM ///**		**immunoglobulin heavy constant gamma 1**	
	**IGHV4-31**		**(G1m marker) /// immunoglobulin heavy**	
			**constant gamma 2 (G2m marker) ///**	
			**immunoglobulin heavy constant mu ///**	
			**immunoglobulin heavy variable 4–31**	
21	IGL@	214677_x_at	immunoglobulin lambda locus	6.3976
		209138_x_at		6.2909
23	JUP /// KRT19	201650_at	junction plakoglobin /// keratin 19	7.8226
	LAMB1	211651_s_at	laminin, beta 1	5.0672
		201505_at		6.4921
*24*	*LBH*	*221011_s_at*	*imb bud and heart development homolog*	*5.5211*
			(*mouse*)	
**25**	**LOC100133662 ///**	**201909_at**	**hypothetical protein LOC100133662 ///**	**−6.9535**
	**RPS4Y1**		**ribosomal protein S4, Y-linked 1**	
26	LPL	203549_s_at	lipoprotein lipase	6.3296
		203548_s_at		6.1435
27	MEST	202016_at	mesoderm specific transcript homolog (mouse)	5.5929
28	MMD	203414_at	monocyte to macrophage	5.4544
			differentiation-associated	
29	MSLN	204885_s_at	mesothelin	5.5812
30	MXRA5	209596_at	matrix-remodelling associated 5	5.3933
31	MYL2	209742_s_at	myosin, light chain 2, regulatory, cardiac, slow	5.8398
*32*	*NPR3*	*219789_at*	*natriuretic peptide receptor C/guanylate cyclase*	*−5.9588*
			*C* (*atrionatriuretic peptide receptor C*)	
33	PCK1	208383_s_at	phosphoenolpyruvate carboxykinase 1 (soluble)	5.7877
34	PFKFB3	202464_s_at	6-phosphofructo-2-kinase/fructose-2,6-biphosph	6.0757
			atase 3	
35	PLA2G2A	203649_s_at	phospholipase A2, group IIA (platelets, synovia	6.9397
			l fluid)	
36	PLIN	205913_at	perilipin	10.0271
37	POMZP3 /// ZP3	204148_s_at	POM (POM121 homolog, rat) and ZP3 fusion ///	5.0854
			zona pellucida glycoprotein 3 (sperm receptor)	
38	PRG4	206007_at	proteoglycan 4	6.4669
39	PRKACA	216234_s_at	protein kinase, cAMP-dependent, catalytic,	−5.7289
			alpha	
40	PSD3	203354_s_at	pleckstrin and Sec7 domain containing 3	−5.4524
**41**	**RBP4**	**219140_s_at**	**retinol binding protein 4, plasma**	**10.2064**
42	RGS1	216834_at	regulator of G-protein signaling 1	8.3498
**43**	**SFRP1**	**202037_s_at**	**secreted frizzled-related protein 1**	**7.4615**
44	SGK1	201739_at	serum/glucocorticoid regulated kinase 1	6.3130
45	SLC16A7	207057_at	solute carrier family 16, member 7	−5.0912
			(monocarboxylic acid transporter 2)	
46	SLPI	203021_at	secretory leukocyte peptidase inhibitor	9.6451
47	SPP1	209875_s_at	secreted phosphoprotein 1	9.1800
48	SULF1	212353_at	sulfatase 1	6.0712
		212354_at		5.7653
49	TF	203400_s_at	transferrin	10.0283
		214063_s_at		6.0445
50	UPK3B	206658_at	uroplakin 3B	6.7799
**51**	**XIST**	**221728_x_at**	**X (inactive)-specific transcript (non-protein**	**8.1499**
		**214218_s_at**	**coding)**	**6.1087**

### The predicted pmAF - related signaling pathways

The DEGs in Table 2 are fed into the KOBAS for predicting the pmAF-related signaling pathways. The KOBAS identified three signaling pathways that satisfy the criterion, as shown in Table 3. We inferred these pathways to be associated with development or maintenance of pmAF.

**Table 3:** The predicted pmAF – related signaling pathways.

No.	Pathway name	The involved DEG	P-value
1	PPAR signaling pathway	ADIPOQ, FABP4, LPL, PLIN, PCK1	5.0E-5
2	Focal adhesion	IGF1, LAMB1, MYL2, SPP1	2.3E-2
3	Dilated cardiomyopathy	IGF1, MYL2, PRKACA	3.3E-2

### Discrimination powers of identified DEGs

The connection relationships among 51 DEGs correspondent to pmAF patients and the calculation of corresponding ROCs and AUCs are respectively shown in Figure S1, Table S1 and S2. The connection network among 51 DEGs (Figure S1) consisted of two subnetworks, four DEG connection pairs and 29 individual DEGs. In two subnetworks, all the DEGs were found to give an AUC of less than 0.7 or more than 0.3 individually (Table S1), which illustrated single DEG had poor discrimination power. When the DEGs in each subnetwork are combined, we calculated the ROC and AUC of each combinational DEGs and found that two AUCs were elevated to 1 (*P* = 0) (Table S2). In 8 DEGs of four pairs, only DICER1 (No. 8) were found to have an AUC of more than 0.7. When these 8 DEGs combined, the AUC was equal to 1 (*P* = 0). 10 of 29 individual DEGs were found to have an AUC of more than 0.7 (Table S1). Among the remaining 19 DEGs, IGF1 (NO.17), MYL2 (No. 31), PCK1 (No. 33) and PRKACA (No. 39) were involved in the pmAF – related PPAR signaling pathways, focal adhesion and dilated cardiomyopathy. When PCK1 was combined with other four DEGs involved in the PPAR pathway, the AUC was more than 0.7 (AUC = 0.926, *P* = 0). Similarly, two AUCs for the combinations of DEGs respectively involved in focal adhesion and dilated cardiomyopathy pathways are 0.826 (*P* = 0.004) and 0.774 (*P* = 0.017). The combination of other 15 individual DEGs was also found to have an AUC of more than 0.7 (AUC = 1, *P = *0).

As have discussed above, different combinations among 51 DEG according to their connection relationships in pmAF samples showed great discrimination power of classifying normal and pmAF samples. This demonstrated that the identified DEGs are reliable and the threshold of identifying DEGs is reasonable.

## Discussion

### The enrichment analysis of identified DEGs

The results for the enrichment analysis of biological process and cellular component on Gene Ontology (GO) and the diseases on Genetic Association Database (GAD) are respectively shown in Table S3, S4 and S5. The existing experimental studies showed that the etiological factors inducing AF mainly include cardiac muscle or organ [1], [11], cardiovascular [2], inflammation [12], [13], proliferation or differentiation [14], fiber/fibrosis [15], [16], external/hormone stimulation [17], [18], extracellular region/matrix [19], [20] and metabolism [5]. 32 of 51 DEGs are included in the statistically enriched GO terms of biological processes, in which 11 are relevant to the cardiac muscle, muscle cell or muscle organ, such as BMP10, RBP4 and MYL2 and other seven DEGs are related to the inflammation, for instance, ADIPOQ, FABP4 and C3; 38 of 51 DEGs are included in the statistically enriched GO terms of cellular component, in which 24 DEGs are located in extracellular region or extracellular matrix (ECM) at the levels of subcellular structures and macromolecular complexes, such as ADIPOQ, BMP10 and IGF1, while others are located in sarcomere, myofibril, contractile fiber and adherens junction; 22 of 51 DEGs are included in the statistically enriched GAD terms of disease, most of which are associated with metabolism and cardiovascular diseases. For example, the ADIPOQ, AMY1A, CFB, HP and HBB are associated with the metabolic diseases, while the FBP4, HP, LPL and MYL2 are related to the cardiovascular diseases.

In order to further illustrate the reliability of identified DEGs, we established the association between the AF-related etiological factors and all the identified DEGs. We firstly connected the factors and the “terms” according to the biological meaning of each term and then established the relationships between the identified DEGs and the etiological factors via the terms in the enrichment analysis results. The 51 DEGs and their association with the AF - related etiological factors are shown in Table S6. The results showed that 37 of 51 DEGs are closely related to the etiological factors inducing AF and so our results have high reliability. Since the pathophysiological mechanisms of AF have not completely been explained, the known factors causing pmAF are not comprehensive. Thus, those genes, such as DIRAS3, HBA1/HBA2, IGH@/IGHA1/IGHA2/IGHV3OR16-13/LOC100126583, MMD, PRKACA and SLC16A7, which do not correlated with any a known etiological factor of AF, may provide new insights for understanding pathophysiological mechanisms of pmAF.

### Analysis of association between the predicted pathways and pmAF

**Figure 2:**
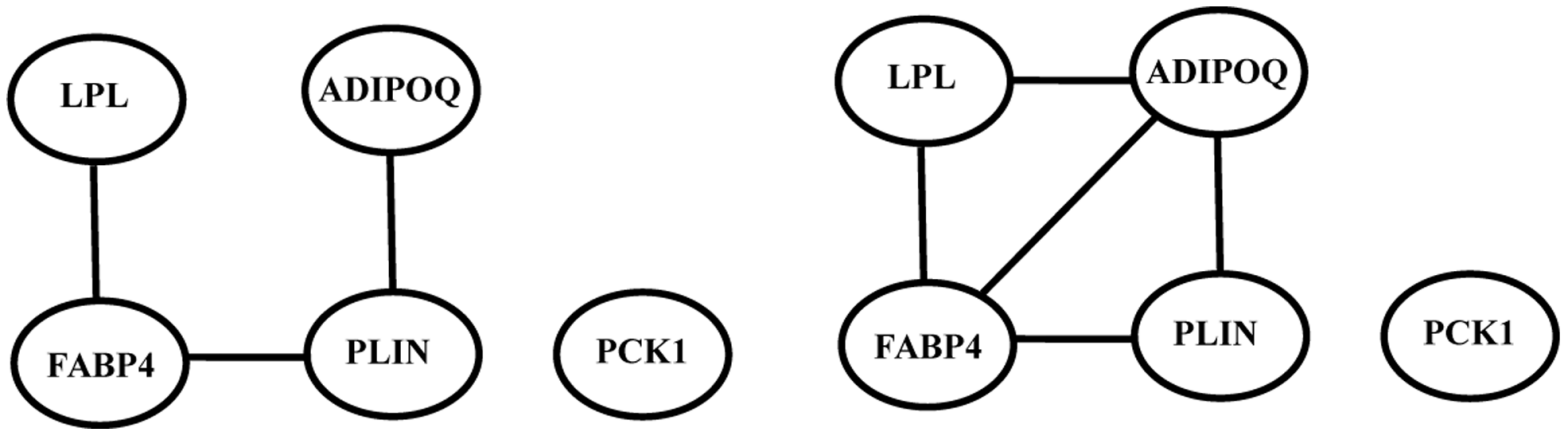
The connection relationships among 5 DEGs in the PPAR signaling pathway. A. The connection relationships in pmAF. B. The connection relationships in controls. The threshold of CC is 0.9.

There are respectively 5, 4, and 3 DEGs in the PPAR, focal adhesion and dilated cardiomyopathy signaling pathways (Table 3). Our previous analysis illustrated that these DEGs are closely associated with pmAF. The abnormal expressions of the DEGs in three predicted signaling pathways are probably one of the reasons that these signaling pathways promote the pmAF progression. Further, using gene expression data in U133A, we analyzed the connections among the DEGs involved in each predicted pathway in AF patients and controls respectively [7]. The connection relationships among five DEGs involved in the PPAR signaling pathway are shown in Figure 2. We found that the connections between ADIPOQ and FABP45 and between ADIPOQ and LPL disappear in pmAF patients (Figure 2(A)), while there are strong pairwise connections among ADIPOQ, FABP4, LPL and PLIN in the controls (Figure 2(B)). The ACK1 is isolated in both cases. The similar results are obtained for the focal adhesion and dilated cardiomyopathy pathways (the data are not given). For instance, in the focal adhesion pathway, the MYL2 and SPP1 interacted in the control (CC = 0.86), but they were not correlated with each other in the pmAF patients (CC = 0.17); although all of the connections among the DEGs in the dilated cardiomyopathy pathway were weak correlation in both pmAF patients and controls, there are great difference between the corresponding CCs in both cases. Thus, we inferred that the alterations of connections among the DEGs in three pathways may be another cause that these signaling pathways promote pmAF.

In addition, some existing researches indirectly supported our prediction. For the PPAR signaling pathway, [21] and [22] illustrated that the peroxisome proliferator-activated receptors (PPARs) are lipid-activated transcription factors that regulate lipid and lipoprotein metabolism, glucose homeostasis, inflammation and cardiovascular system; The PPARs are a family of three nuclear hormone receptors, PPARα, -β/δ, and –γ, in which the PPARγ activator pioglitazone can attenuate congestive heart failure-induced atrial structural remodeling and AF promotion, with effects similar to those of candesartan [15]. The focal adhesions are large multi-protein assemblies that form at the basal surface of cells on planar dishes, and that mediate cell signaling, force transduction and adhesion to the substratum [23]. The modulation of focal adhesion assembly/disassembly in response to mechanical load may be related to a primary role for focal adhesion assembly in myofibrillogenesis [24]. Like their costameric counterparts in vivo, the cardiomyocyte focal adhesions contain vinculin and other cytoskeletal proteins that form a dense adhesion plaque at sites of close approximation of the sarcolemma to the ECM. The increase in cardiomyocyte ECM deposition results in abnormal conduction through the atria, thus creating a substrate for atrial fibrillation [25]. The Dilated cardiomyopathy (DCM), a genetically heterogeneous disorder, causes heart failure and rhythm disturbances. The dilated cardiomyopathy was typically preceded by atrial fibrillation, sinus node dysfunction, and conduction block [26]. Remodeling occurs in both ventricle and atrium in dilated cardiomyopathy. Thus, the dilated cardiomyopathy might cause pmAF by the alteration of atrial ECM components during remodeling [20].

### Comparison between the APCA and other related methods

**Figure 3:**
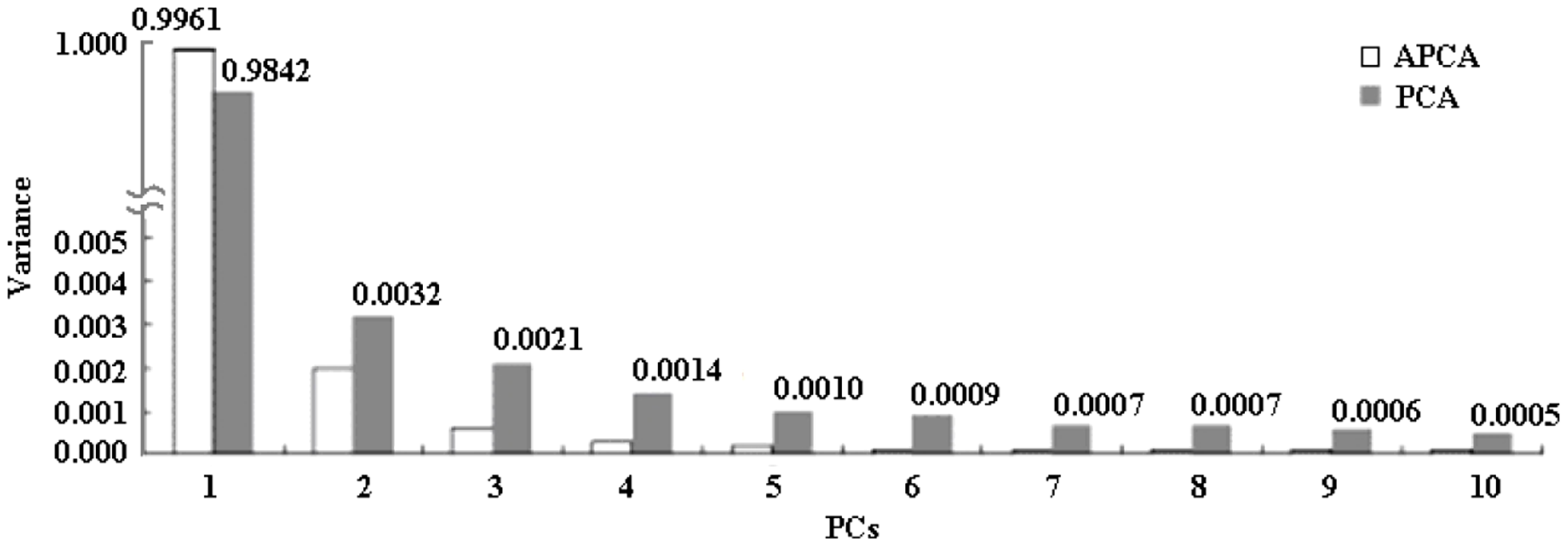
The first 10 PCs extracted by APCA and PCA [6].

The study of Censi, et al. [6] illustrated the effectiveness and feasibility of PCA method in finding disease –related biological features. APCA is an improved PCA and both have same theoretical basis. Therefore we first compare APCA with PCA. Figure 3 shows the first 10 PCs extracted by APCA and PCA respectively. Their first PCs respectively account for 99.61% and 98.42%. In minor PCs, the second PC of APCA is much larger than the third PCs onward, while the second PC of PCA is comparable with the third to the fifth PCs. Our simulation showed that PCA is undesirable or has drawbacks for the data analysis with different numbers of samples in the different classes because PCA uses the number of the samples to weight the class conditional covariance matrix in constructing the total scatter matrix. As such, the class with large number of samples will dominate the results of the principle components of PCA while the information of the class with small number of samples cannot be well shown in its principal components. Now the APCA takes *a* = 0.3 and so the larger weight ((1-*a*) = 0.7 comparing to 0.345 (10/29) of PCA) is used for the class of pmAF. Thus, information of the class of pmAF is emphasized in APCA (0.7>0.5) while it is deemphasized in PCA (0.345<0.5). Furthermore, with *b* = 20 (it is significantly larger than *b* = 1 in PCA), APCA forces the largest PC to capture the difference of the class means and hence clearly separates the information about the difference of the class means from the information about the within-class variations into different principal components. PCA with *b* = 1 makes these two different types of information mixed in various PCs. Thus, the first two PCs of APCA have higher discriminating power of classifying normal and pmAF samples than that of PCA since APCA considers the unbalanced sample numbers.

Numerous feature selection methods have been applied to the identification of DEGs on microarray, including Fold change, Welch t-statistic, SAM (Significance Analysis of Microarray), etc. [27]. The feature selection methods separately identify each DEG that has significant difference in statistics and the number of identified DEGs is usually very large, while APCA identify DEGs whose expressions are correlated. Since the AF signature is activated by a general modulation of the whole genome but a single gene, APCA is able to better characterize different pathophysiological aspects of AF. Typically, the number of samples is limited by the availability of sufficient patients or cost and the noise is inevitable in a microarray study. The number of samples and noise are significant challenge to any feature selection approaches [27], while APCA is more robust to both factors [28]. For a microarray data with unbalanced samples, APCA is able to allocate larger weight to the group with fewer sample number for reducing the influence of imbalance on the final results. Therefore APCA can produce more reliable results than other methods that do not consider the problem of unbalanced sample number when processing U133A dataset, which is a typical microarray data with unbalanced samples.

### Comparing with the existing results

By PCA, Censi, et al. identified 50 pmAF - related DEGs from the same data set [6]. APCA and PCA' mechanisms of weighting two classes of samples (pmAF and control) are very different so that the scores of same a gene generated by APCA and PCA are very different. Therefore, APCA and PCA identify different DEG lists that have very low overlap. This is the main reason why only 6 genes are same between two DEG lists identified by our and Censi, et al.'s methods.

Our enrichment analysis about biological process and cellular component on GO for 50 DEGs also shows the majority of them (27 DEGs, while ours is 37 DEGs) are individually related to the etiological factors inducing AF. Using 50 DEGs extracted by Censi, et al., we do not find any a gene is included in the statistically enriched GAD terms of disease on GAD (we have 22 DEGs), and only one statistically enriched pathway named focal adhesion is found on KOBAS, in which genes JUN, PIK3R1, TNC and THBS4 are involved. This illustrates that the correlation in biological functions among our 51 DEGs is higher than that of 50 DEGs. Therefore, there are more genes and combinational works of multiple genes in our 51 DEGs to be associated with occurrence and progress of pmAF. APCA is a more appropriate method to microarray data that have unbalanced samples.

Finally, it is worthy explaining that we do not analyze the U133B data set because too many genes were not annotated on this chip, which may result in wrong interpretation to the final results. The pathophysiology of pmAF is extremely complex. In our future work, we shall validate the suggested pmAF-related DEGs in experiments and integrate multiple types of data (such as gene sequence, RNA and miRNA expression profiles, protein-protein interactions) to build functional networks promoting pmAF for more comprehensive understanding of pmAF pathophysiology.

## Conclusion

This work proposes a novel method to identify the DEGs from microarray data with unbalanced sample numbers. 51 DEGs associated with pmAF are identified, in which 42 DEGs are different from the existing related results. The PPAR, focal adhesions and dilated cardiomyopathy signaling pathways are predicted to be associated with pmAF based on all of the identified DEGs. This work provides some new insights into biological features of pmAF and has also the potentially important implications for improved understanding of the molecular mechanisms of pmAF.

## Supporting Information

Figure S1 **The connection network among 51 identified DEGs.** The No. of each DEG is same with that in Table 2.
(TIF)

Table S1 The AUCs of 51 DEGs individually.
(DOC)

Table S2 The AUCs of combination among multiple genes.
(DOC)

Table S3 The statistically enriched GO terms of biological processes.
(XLS)

Table S4 The statistically enriched GO terms of cellular component.
(XLS)

Table S5 The statistically enriched GAD terms of disease.
(XLS)

Table S6 The association between the identified DEGs and the etiological factors inducing pmAF.
(DOC)
