# Supplementary figures and images for: Analysis on Differential Gene Expression Data for Prediction of New Biological Features in Permanent Atrial Fibrillation
Source: PLoS One. 2013 Oct 18;8(10):e76166. doi: 10.1371/journal.pone.0076166 (PMC3799783; doi:10.1371/journal.pone.0076166)

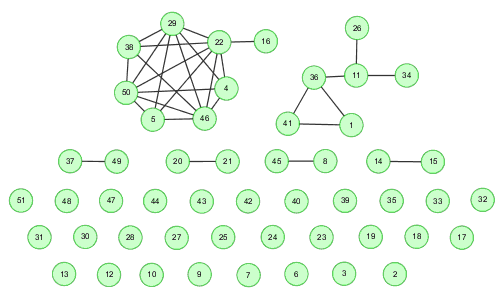

Supplement: Figure S1 — The connection network among 51 identified DEGs. The No. of each DEG is same with that in Table 2. (TIF) [file pone.0076166.s001.tif]
