# Supplementary material for: Analysis on Differential Gene Expression Data for Prediction of New Biological Features in Permanent Atrial Fibrillation
Source: PLoS One. 2013 Oct 18;8(10):e76166. doi: 10.1371/journal.pone.0076166 (PMC3799783; doi:10.1371/journal.pone.0076166)
Supplement: Table S1 — The AUCs of 51 DEGs individually. (DOC) [file pone.0076166.s002.doc]

Table S1. The AUCs of 51 DEGs individually

| **No.** | **Gene symbol** | **AUC** | **P_Value** |
| --- | --- | --- | --- |
| 1 | ADIPOQ | ***0.474*** | ***0.819*** |
| 2 | AMY1A /// AMY1B /// AMY1C | ***0.337*** | ***0.155*** |
|  | /// AMY2A /// AMY2B |  |  |
| 3 | BMP10 | ***0.189*** | ***0.007*** |
| 4 | C2 /// CFB | ***0.516*** | ***0.891*** |
| 5 | C3 | ***0.421*** | ***0.491*** |
| 6 | CEBPA | ***0.311*** | ***0.099*** |
| 7 | COL21A1 | ***0.832*** | ***0.004*** |
| 8 | DICER1 | ***0.179*** | ***0.005*** |
| 9 | DIRAS3 | ***0.842*** | ***0.003*** |
| 10 | EFEMP1 | ***0.647*** | ***0.199*** |
| 11 | FABP4 | ***0.595*** | ***0.409*** |
| 12 | FHL2 | ***0.616*** | ***0.313*** |
| 13 | GOLGA8A | ***0.668*** | ***0.142*** |
| 14 | HBA1 /// HBA2 | ***0.542*** | ***0.714*** |
| 15 | HBB | ***0.468*** | ***0.783*** |
| 16 | HP /// HPR | ***0.479*** | ***0.854*** |
| 17 | IGF1 | ***0.426*** | ***0.521*** |
| 18 | IGFBP2 | ***0.932*** | ***0.000*** |
| 19 | IGH@ /// IGHA1 /// IGHA2 /// | ***0.463*** | ***0.748*** |
|  | IGHV3OR16-13 /// |  |  |
|  | LOC100126583 |  |  |
| 20 | IGH@ /// IGHG1 /// IGHG2 /// | ***0.489*** | ***0.927*** |
|  | IGHM /// IGHV4-31 |  |  |
| 21 | IGL@ | ***0.537*** | ***0.748*** |
| 22 | JUP /// KRT19 | ***0.516*** | ***0.891*** |
| 23 | LAMB1 | ***0.721*** | ***0.054*** |
| 24 | LBH | ***0.863*** | ***0.002*** |
| 25 | LOC100133662 /// RPS4Y1 | ***0.484*** | ***0.891*** |
| 26 | LPL | ***0.542*** | ***0.714*** |
| 27 | MEST | ***0.521*** | ***0.854*** |
| 28 | MMD | ***0.379*** | ***0.291*** |
| 29 | MSLN | ***0.505*** | ***0.963*** |
| 30 | MXRA5 | ***0.847*** | ***0.002*** |
| 31 | MYL2 | ***0.500*** | ***1.000*** |
| 32 | NPR3 | ***0.153*** | ***0.002*** |
| 33 | PCK1 | ***0.474*** | ***0.819*** |
| 34 | PFKFB3 | ***0.474*** | ***0.819*** |
| 35 | PLA2G2A | ***0.468*** | ***0.783*** |
| 36 | PLIN | ***0.468*** | ***0.783*** |
| 37 | POMZP3 /// ZP3 | ***0.563*** | ***0.582*** |
| 38 | PRG4 | ***0.653*** | ***0.183*** |
| 39 | PRKACA | ***0.463*** | ***0.748*** |
| 40 | PSD3 | ***0.268*** | ***0.044*** |
| 41 | RBP4 | ***0.495*** | ***0.963*** |
| 42 | RGS1 | ***0.537*** | ***0.748*** |
| 43 | SFRP1 | ***0.395*** | ***0.359*** |
| 44 | SGK1 | ***0.568*** | ***0.551*** |
| 45 | SLC16A7 | ***0.316*** | ***0.108*** |
| 46 | SLPI | ***0.568*** | ***0.551*** |
| 47 | SPP1 | ***0.800*** | ***0.009*** |
| 48 | SULF1 | ***0.468*** | ***0.783*** |
| 49 | TF | ***0.500*** | ***1.000*** |
| 50 | UPK3B | ***0.547*** | ***0.680*** |
| 51 | XIST | ***0.384*** | ***0.313*** |
