# Supplementary material for: Analysis on Differential Gene Expression Data for Prediction of New Biological Features in Permanent Atrial Fibrillation
Source: PLoS One. 2013 Oct 18;8(10):e76166. doi: 10.1371/journal.pone.0076166 (PMC3799783; doi:10.1371/journal.pone.0076166)
Supplement: Table S2 — The AUCs of combination among multiple genes. (DOC) [file pone.0076166.s003.doc]

Table S2. The AUCs of combination among multiple genes

| **Combination** | **The involved DEGs** | **AUC** | **P_value** |
| --- | --- | --- | --- |
| **Subnetworks** | 4,5,16,22,29,38,46,50 | 1 | 0 |
|  | 1,11,26,34,36,41 | 1 | 0 |
| **DEG pairs** | 8,45;14,15;20,21;37,49 | 1 | 0 |
| **PPAR signaling pathway** | 1,11,26,33,36 | 0.926 | 0 |
| **Focal adhesion** | 17,23,31,47 | 0.826 | 0.004 |
| **Dilated cardiomyopathy** | 17,31,39 | 0.774 | 0.017 |
| **15 single-genes** | 2,6,10,12,13,19,25,27,28,35,42, | 1 | 0 |
|  | 43,44,48,51 |  |  |
