# Supplementary material for: Analysis on Differential Gene Expression Data for Prediction of New Biological Features in Permanent Atrial Fibrillation
Source: PLoS One. 2013 Oct 18;8(10):e76166. doi: 10.1371/journal.pone.0076166 (PMC3799783; doi:10.1371/journal.pone.0076166)
Supplement: Table S6 — The association between the identified DEGs and the etiological factors inducing pmAF. (DOC) [file pone.0076166.s007.doc]

| Table S6. The association between the identified DEGs and the etiological factors inducing pmAF | | | | | | | | | |
| --- | --- | --- | --- | --- | --- | --- | --- | --- | --- |
| **No.** | **Gene** | **A** | **B** | **C** | **D** | **E** | **F** | **G** | **H** |
| 1 | ADIPOQ | √ | √ | √ | √ |  | √ | √ | √ |
| 2 | AMY1A /// AMY1B /// AMY1C /// |  |  |  |  |  |  | √ | √ |
|  | AMY2A /// AMY2B |  |  |  |  |  |  |  |  |
| 3 | BMP10 | √ |  |  | √ | √ |  | √ |  |
| 4 | C2 /// CFB | √ |  | √ |  |  | √ | √ | √ |
| 5 | C3 |  |  | √ |  |  | √ | √ |  |
| 6 | CEBPA |  |  |  | √ |  |  |  |  |
| 7 | COL21A1 |  |  |  |  |  |  | √ |  |
| 8 | DICER1 | √ |  |  | √ |  |  |  |  |
| 9 | DIRAS3 |  |  |  |  |  |  |  |  |
| 10 | EFEMP1 |  |  |  |  |  |  | √ |  |
| 11 | FABP4 | √ | √ | √ | √ |  | √ |  |  |
| 12 | FHL2 | √ |  |  | √ | √ | √ |  |  |
| 13 | GOLGA8A |  |  |  |  |  |  |  |  |
| 14 | HBA1 /// HBA2 |  |  |  |  |  |  |  |  |
| 15 | HBB |  | √ |  |  |  |  |  | √ |
| 16 | HP /// HPR |  | √ |  |  |  |  | √ | √ |
| 17 | IGF1 |  | √ |  | √ |  |  | √ | √ |
| **18** | **IGFBP2** | √ |  |  |  |  | √ | √ |  |
| 19 | IGH@ /// IGHA1 /// IGHA2 /// |  |  |  |  |  |  |  |  |
|  | IGHV3OR16-13 /// LOC100126583 |  |  |  |  |  |  |  |  |
| **20** | **IGH@ /// IGHG1 /// IGHG2 /// IGHM** |  |  |  |  |  |  | √ |  |
|  | **/// IGHV4-31** |  |  |  |  |  |  |  |  |
| 21 | IGL@ |  |  |  |  |  |  | √ |  |
| 22 | JUP /// KRT19 |  |  |  |  | √ |  |  |  |
| 23 | LAMB1 |  |  |  | √ |  |  | √ |  |
| 24 | LBH |  |  |  |  |  |  |  |  |
| **25** | **LOC100133662 /// RPS4Y1** |  |  |  |  |  |  |  |  |
| 26 | LPL |  | √ |  | √ |  |  | √ | √ |
| 27 | MEST |  |  |  |  |  | √ |  |  |
| 28 | MMD |  |  |  |  |  |  |  |  |
| 29 | MSLN |  |  |  |  |  |  | √ |  |
| 30 | MXRA5 |  |  |  |  |  |  | √ |  |
| 31 | MYL2 | √ | √ |  |  | √ |  |  |  |
| 32 | NPR3 |  | √ |  |  |  |  |  |  |
| 33 | PCK1 | √ | √ |  |  |  | √ |  | √ |
| 34 | PFKFB3 |  |  |  |  |  |  |  |  |
| 35 | PLA2G2A |  | √ | √ | √ |  | √ | √ | √ |
| 36 | PLIN |  |  |  |  |  |  |  | √ |
| 37 | POMZP3 /// ZP3 |  |  |  |  |  |  |  |  |
| 38 | PRG4 |  |  |  |  |  |  | √ |  |
| 39 | PRKACA |  |  |  |  |  | √ |  |  |
| 40 | PSD3 |  |  |  |  |  |  |  |  |
| **41** | **RBP4** | √ |  |  | √ |  | √ | √ | √ |
| 42 | RGS1 |  |  |  |  |  |  |  |  |
| **43** | **SFRP1** |  |  |  |  |  |  | √ |  |
| 44 | SGK1 |  | √ |  |  |  |  |  |  |
| 45 | SLC16A7 |  |  |  |  |  |  |  |  |
| 46 | SLPI |  |  |  |  |  |  | √ |  |
| 47 | SPP1 | √ | √ | √ | √ |  | √ | √ | √ |
| 48 | SULF1 |  |  |  |  |  |  | √ |  |
| 49 | TF |  | √ | √ |  |  |  | √ | √ |
| 50 | UPK3B |  |  |  |  |  |  |  |  |
| **51** | **XIST** |  |  |  |  |  |  |  |  |

**Note: A -** cardiac muscle or organ; **B** – cardiovascular; **C** – inflammation; **D** - proliferation or differentiation; **E** - fiber/fibrosis; **F** - external/hormone stimulation; **G** - extracellular region/matrix; **H** - metabolism.
